# Piezoelectric fibers for flexible and wearable electronics

**DOI:** 10.1007/s12200-023-00058-3

**Published:** 2023-03-22

**Authors:** Shengtai Qian, Xingbei Wang, Wei Yan

**Affiliations:** 1 State Key Laboratory for Modification of Chemical Fibers and Polymer Materials, College of Materials Science and Engineering, Donghua University, Shanghai, 201620 China; 2 School of Electrical and Electronic Engineering, Nanyang Technological University, Singapore, 639798 Singapore; 3 School of Materials Science and Engineering, Nanyang Technological University, Singapore, 639798 Singapore

**Keywords:** Piezoelectric materials and devices, Flexible electronics, Smart fibers, Intelligent fabrics

## Abstract

Flexible and wearable electronics represent paramount technologies offering revolutionized solutions for medical diagnosis and therapy, nerve and organ interfaces, fabric computation, robot-in-medicine and metaverse. Being ubiquitous in everyday life, piezoelectric materials and devices play a vital role in flexible and wearable electronics with their intriguing functionalities, including energy harvesting, sensing and actuation, personal health care and communications. As a new emerging flexible and wearable technology, fiber-shaped piezoelectric devices offer unique advantages over conventional thin-film counterparts. In this review, we survey the recent scientific and technological breakthroughs in thermally drawn piezoelectric fibers and fiber-enabled intelligent fabrics. We highlight the fiber materials, fiber architecture, fabrication, device integration as well as functions that deliver higher forms of unique applications across smart sensing, health care, space security, actuation and energy domains. We conclude with a critical analysis of existing challenges and opportunities that will be important for the continued progress of this field.

**Graphical Abstract:**

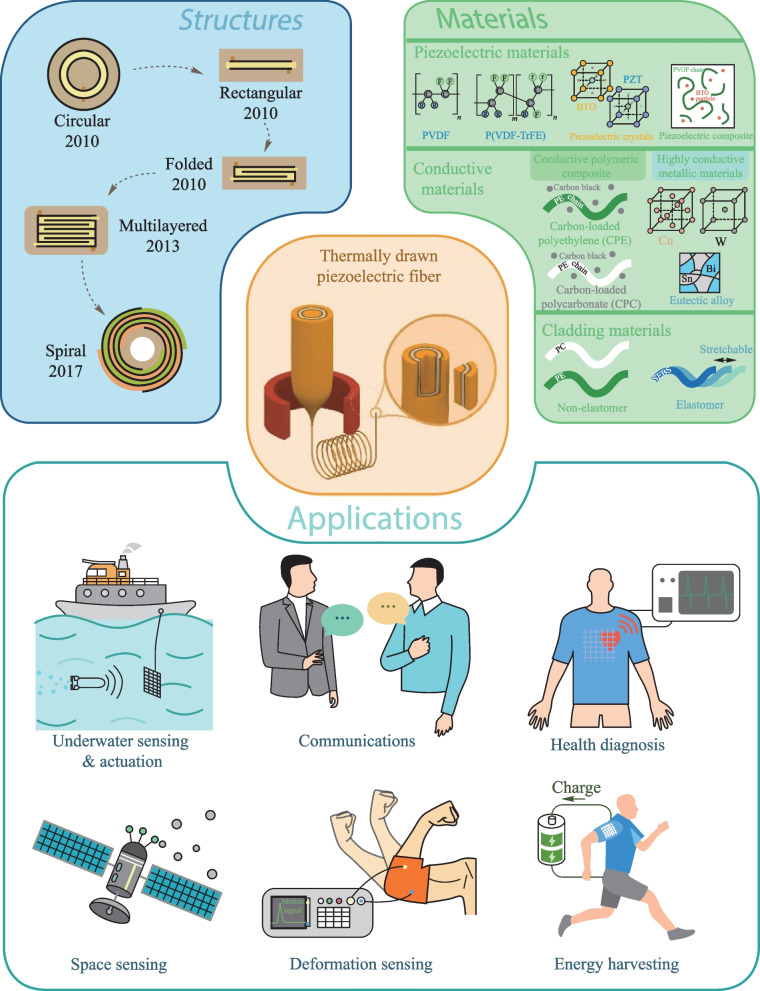

## Introduction

Flexible and wearable electronics, an innovative variant of conventional rigid electronics, represents powerful technologies for a myriad of applications in healthcare, energy, sustainability, and neuroscience [1–20]. For such capabilities, many recent flexible and wearable technologies leverage piezoelectric materials and devices for mechanical and electrical energy management, thereby enabling smart sensing, actuation and energy harvesting [21–23]. Typical approaches to flexible and wearable piezoelectric device fabrication rely on the integration of thin piezoelectric materials onto flexible thin-film substrates [24, 25]. Despite wide-ranging potential applications, thin-film-based piezoelectric device technology suffers from some limitations. First, many devices use off-the-shelf piezo-products without innovation in materials or properties, which limits device performance. Secondly, the fabrication usually involves multiple complex steps, restricting upscaling. Thirdly, the devices are limited to some specific planar form factors, which are neither breathable nor comfortable. Interfacing such devices with human body adds much of a burden to the potential wearers. All these issues frustrate the practical and widespread usage of these devices.

The recent development of fiber-shaped piezoelectric devices has emerged as an innovative flexible and wearable platform [26–31]. Various fiber fabrication methods such as melting spinning, jet spinning, electrospinning, dip coating, chemical and physical deposition, electrochemical deposition, and hydrothermal synthesis have been developed [4, 30–34]. However, these techniques require tedious and complicated procedures to fabricate fibers, and the resulting fibers are limited to simple architecture and functionalities. Among the numerous fiber technologies, the preform-to-fiber thermal drawing holds special promise for both novel fundamental science research and unique practical device applications [35–48]. This approach exploits the thermal drawing of a macroscopic preform that integrates a variety of functional materials with different physical and chemical properties. Engineering materials at both the microscale and nanoscale during thermal drawing enable unusual fiber devices with excellent performance. The preform is constructed at the centimeter-scale, and thus, complex cross-sectional architecture can be easily achieved [49, 50]. The subsequent thermal drawing produces ultralong fibers with sophisticated geometries in a single step, which makes this approach particularly useful for large-scale manufacturing. Moreover, the small cross section and high aspect ratio allow the fibers to be woven into fabrics, delivering a new generation of smart wearables that offer value-added service for society [33, 51].

**Fig. 1:**
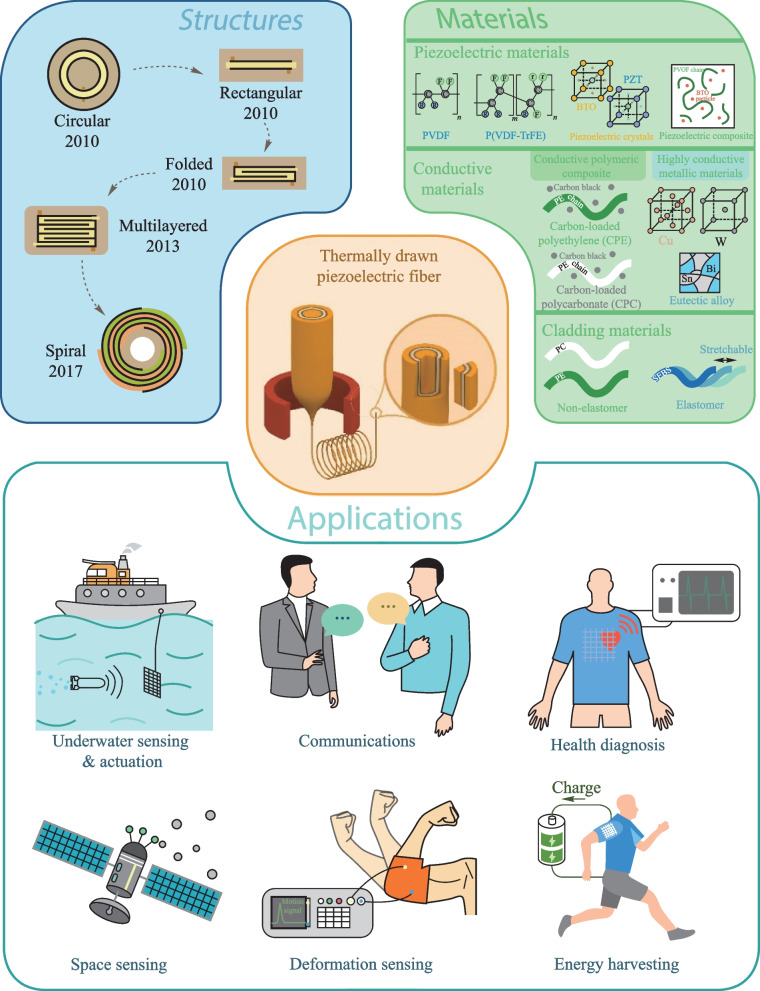
Summary of the structures, materials and applications of the thermally-drawn piezoelectric fibers (TDPFs)

Recent advance in thermally-drawn piezoelectric fibers epitomizes their capabilities of addressing a series of challenges in flexible and wearable electronics. In this review, we highlight scientific and technological breakthroughs in thermally-drawn piezoelectric fibers (TDPFs), focusing on fiber materials and structures, fabrication process, and applications, as shown in Fig. 1. Section 2 introduces the fabrication principles of TDPFs. Typical materials used for TDPFs and various recently developed fiber architectures are then discussed. In the following section, we highlight the new generation of piezoelectric devices that deliver wide-ranging applications in underwater sensing, acoustic sensing and communication, physiologic sensing, space dust sensing and energy harvesting. Finally, we conclude the review with some viewpoints about the challenges and opportunities faced by TDPFs that will further trigger the development of the field.

## Thermal drawing

### Basic principles

**Fig. 2:**
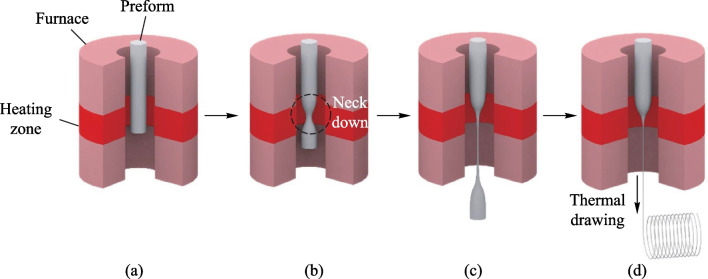
Schematic representation of the thermal drawing process

Thermal drawing was traditionally adopted for the production of optical fibers made of a single material, with the ability to produce fibers with tunable diameters and extremely long lengths at a high manufacturing rate [52]. Innovations in materials and fabrication methodologies create a new generation of thermally-drawn multi-material fiber electronics and optoelectronics. Thermal drawing begins with the construction of a preform, a scaled-up model of the desired fiber that has a pre-designed transversal structure (Fig. 2a). The structure usually stays unaltered during thermal drawing, thereby producing fibers that maintain the same materials and architecture. The preform cladding is usually made of polymers or polymer-based composites, which are highly plastic during the drawing process. Various preform fabrication approaches have been reported, including thin-film rolling, deep-hole drilling, casting, direct assembly and extrusion [35]. The quality of the preform largely affects the quality of TDPF products. The prepared preform is then fed downward into a vertically positioned tube furnace for thermal drawing. After being soaked in the hot zone for a sufficiently long period, the preform forms a neck-down region under the impact of temperature and external force. (Fig. 2b). The bottom part of the preform then continues to drop down, forming a thin fiber (Fig. 2c). Finally attaching the formed fiber with the pulling system enables fiber production in a continuous and controllable manner. The fiber size can be tuned by the thermal drawing parameters, such as drawing temperature *T*_D_, drawing speed *v*_d_, and preform feeding speed *v*_f_. Based on the volume conservation principle, namely the preform volume fed into the furnace is equal to the fiber volume drawn out of the furnace, the drawing speed *v*_d_, the preform feeding speed *v*_f_, and the perform diameter dictate the fiber diameter:

1 $${D}_{\mathrm{fiber}}={D}_{\mathrm{preform}}\times \sqrt{\frac{{v}_{\mathrm{f}}}{{v}_{\mathrm{d}}}},$$

where *D*_fiber_ is the diameter of the thermally-drawn fiber, *D*_preform_ is the diameter of the preform, *v*_f_ is the preform feeding speed, and *v*_d_ is the drawing speed of fiber.

This rule helps us to obtain desired fibers with tunable diameters ranging from millimeter-scale to microscale by simple control over the drawing parameters. Other factors including materials viscosity, thermal expansion coefficient, chemical stability and surface tension also affect the thermal drawing process. We refer the readers to other relevant publications [53, 54].

### Criteria for material selection

Thermal drawing of multi-material fibers involves materials with disparate physical and chemical properties. Some criteria should be met to ensure that the obtained fiber maintains the identical cross-sectional architecture as the preform. First, the cladding materials should exhibit relatively high viscosity to support the drawing stress and encapsulate all materials inside the fiber. Secondly, the viscosities of different materials should be compatible to achieve synergetic thermoplastic deformation or thermal flow during drawing. Thirdly, when crystalline materials, such as low melting point electrode materials, are integrated, their melting points should be below the thermal drawing temperature to ensure their stable flow. The materials interfacing with the crystalline materials should be in a highly viscous state to avoid mutual mixing. Lastly, the thermal expansion coefficients of all materials should be within a small range at the drawing temperature to prevent the formation of fractures and cracks at the interface [9].

## Materials and structures of thermally drawn piezoelectric fibers (TDPFs)

**Fig. 3:**
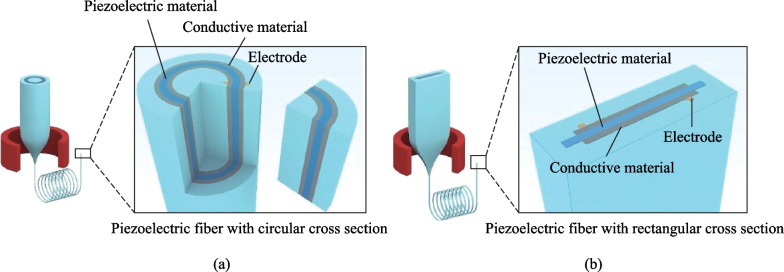
Schematic representations of thermally drawn piezoelectric fibers with **a** circular structure and **b** rectangular structure

TDPF devices consist of piezoelectric materials, conductive electrodes and encapsulating materials. Some typical designs are depicted in Fig. 3, where the piezoelectric material is sandwiched between two layers of polymeric electrodes in contact with a metallic electrode respectively and the whole assembly is encapsulated in a protective cladding. The piezoelectric material, acting as the primary functional domain, converts mechanical deformations or vibrations into electrical signals or vice versa. The conductive materials are placed on both sides of the piezoelectric material to either measure the voltage when mechanical energy is converted into electrical energy or apply an electric field when electrical energy is converted into mechanical energy [55]. In the next sections, we will elaborate the typical piezoelectric materials and device architecture of TDPFs.

### Materials

#### Piezoelectric materials

**Fig. 4:**
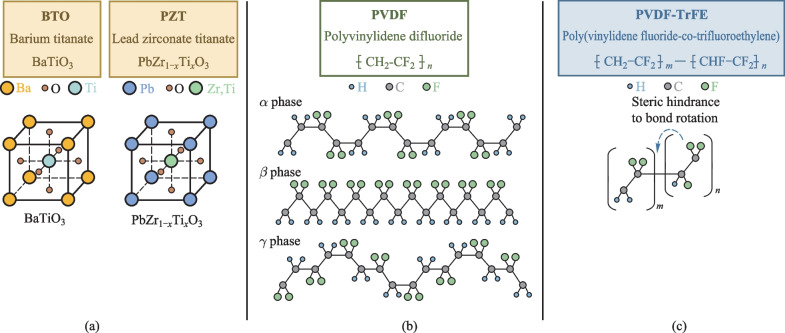
Molecule structures of **a** BTO, PZT, **b** PVDF and **c** P(VDF-TrFE)

Some typical piezoelectric materials include ceramic barium titanate (BaTiO_3_, BTO) and lead zirconate titanate (PbZr_1−*x*_Ti_*x*_O_3_, PZT) [56]. BTO and PZT are ABO_3_ perovskite materials with cubic crystal structure below Curie temperature *T*_c_ (Fig. 4a). Both would undergo phase changes above their respective Curie temperature, so their working and processing temperature must not exceed the Curie temperature [24, 57, 58].

Although BTO- and PZT-based piezoelectric materials have been widely adopted in planar piezoelectric devices because of their extraordinary performances, their bulky forms cannot be directly used as the main piezoelectric materials in TDPFs, because of their high melting points incompatible with the thermal drawing process. Instead, polymeric polyvinylidene difluoride (PVDF) and poly(vinylidene fluoride-co-trifluoroethylene) (P(VDF-TrFE)) are the main piezoelectric materials used to construct TDPF devices due to their low processing temperatures and excellent piezoelectric properties [59].

PVDF is the first discovered polymeric material that exhibits strong piezoelectricity [60]. Such piezoelectricity is closely related to its molecular structure (CH_2_-CF_2_)_*n*_. The strong electronegativity difference between C and F atoms leads to a strong polarity. The piezoelectric performance of PVDF largely depends on the orientation of these C-F bonds. A strict alignment in the same direction of all C-F bonds is nearly impossible since two C-F bonds in a monomer share a common carbon atom in PVDF, which causes the inevitable deviation of bond orientations.

However, in certain crystal forms of PVDF, the orientations of C-F bonds are largely the same, generating strong piezoelectricity. There are three types of crystalline phases of PVDF, denoted as α (form II), β (form I), γ (form III) [61]. Figure 4b shows the molecular configuration of each phase. These crystalline phases usually coexist with amorphous phase in the materials. β phase follows a zigzag molecular chain conformation (TTTTTT…), demonstrating the strongest piezoelectricity since all C-F bonds are aligned toward one side of the molecular chain. α phase has a spiral molecular chain conformation (TGTG’TGTG’…), with F atoms alternatively appearing on each side around the chain. The entire molecule chain does not show polarity [62]. α phase is usually the major component of pre-processed PVDF, since it is more thermodynamically stable. The γ phase is much rarer in PVDF and usually appears only when the fabrication process involves certain organic solvents (DMSO, DMA) [63]. The molecular chain conformation of the γ phase (TTTGTTTG’…) is less regular than the β phase, and therefore its piezoelectricity is weak [64]. Therefore, a common approach to obtain PVDF materials with high piezoelectricity is to increase their β phase contents. Mechanically rolling or stretching, either uniaxially or biaxially, has been proven effective to convert α phase to β phase [65]. Other approaches such as electrical treatment have also been reported effective in enhancing PVDF piezoelectricity [66].

P(VDF-TrFE) is a more commonly used piezoelectric material. Unlike PVDF, P(VDF-TrFE) has only one crystalline phase variant due to the steric hindrance to the rotation of the C–C bond caused by the extra F atom (Fig. 4c). Enhancement of piezoelectricity of P(VDF-TrFE) could thus be achieved by increasing its crystallinity, which greatly simplifies the pre-treatment of P(VDF-TrFE) [67]. However, compared to PVDF, raw P(VDF-TrFE) is more costly, which would also increase the cost of TDPF devices.

The piezoelectricity of PVDF and P(VDF-TrFE) can be enhanced by loading piezoelectric ceramic particles (BTO and PZT) [68], or carbon-based materials (carbon nanotubes) [69]. However, it should be noted that these particles could deteriorate the flexibility of TDPFs. The filler percentage needs to be carefully adjusted to balance the piezoelectric and mechanical performance of the polymeric piezocomposites.

#### Conductive materials

Conductive materials are of crucial importance to the TDPFs. They are used to measure the voltage for sensing or provide an electric field for actuation. Conductive materials in the thermally-drawn fiber platform can be classified into two groups: conductive polymeric composites, and highly conductive metallic electrodes.

Conductive polymeric composites (e.g., carbon-loaded polycarbonate (CPC), carbon-loaded polyethylene (CPE)) should intimately interface with the piezoelectric material. During the thermal drawing process, the conductive polymer layers form large-area electrodes and can help to protect and maintain the shape of piezoelectric polymers that are susceptible to capillary break-up at low viscosity molten state [70, 71]. Therefore, ideal conductive polymeric composites should exhibit an excellent combination of thermal plasticity, appropriate viscosity, and good conductivity. The conductivity of these polymeric electrodes is tunable depending on the loaded conductive fillers.

**Fig. 5:**
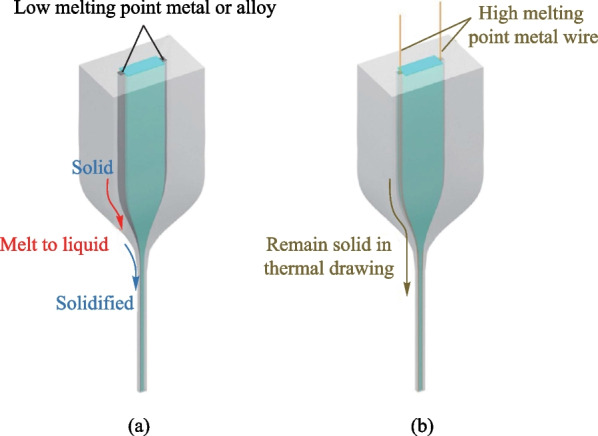
Illustrations of sending electrodes into fibers in the thermal drawing process. **a** Low melting point metals or alloys, **b** high melting point metal wires

Because of the relatively low electrical conductivity of the polymeric electrodes, highly conductive metallic electrodes are always used in TDPFs to interface with the polymeric electrode. Two different metallic electrodes have been reported. The first type utilizes low melting point metals or alloys, like In (Indium), Sn (tin) or eutectic alloys (BiSn) [55, 71–73]. These materials melt into viscous fluids at the neck-down region, and, thus, they can undergo stable thermoplastic deformation and thermal-flow (Fig. 5a). These electrodes are always embedded into the polymeric electrode matrix, forming a highly conductive composite electrode. Their sizes are tunable depending on the draw-down ratio. Thus, ultrasmall metallic electrodes down to the feature sizes of a few micrometers can be achieved, which does not affect the flexibility and wearability of the fiber. The second type of electrodes relies on high-melting-temperature metals such as Cu (copper) and W (tungsten) [74, 75]. These metal wires remain in the solid state during the drawing process and are fed into the predesigned channels, where they undergo thermoplastic deformation and are scaled down to exactly the same size as the metallic wires (Fig. 5b). Despite a simpler process, the metallic electrodes are usually of larger sizes, which might impair the fiber flexibility and wearability.

#### Cladding materials

The cladding usually serves as a protection layer for the inner domains of the fiber. The claddings are usually made of electrical insulating polymers, such as polycarbonate (PC), polysulfone (PSU), or poly(styrene-b-(ethylene-co-butylene)-b-styrene (SEBS) [76]. PC exhibits good optical properties such as high transparency and is widely used in polymeric optical fibers. PSU has a relatively higher glass transition temperature *T*_g_ and is especially used to construct fibers with improved mechanical robustness. SEBS possesses excellent elasticity at room temperature and is frequently used to construct elastic fibers [77, 78]. It should be noted that the viscosity and thermal expansion coefficient of the cladding materials should be carefully identified to ensure the co-drawing of multiple materials.

### Structures

The simplest structure of the piezoelectric fiber relies on the conductor- piezoelectric material-conductor sandwich either in a rectangular or circular form. Early designs of TDPFs share a common drawback, namely the conductive-piezoelectric interface area, referred to as the active area, is relatively small. The limited active area produces insufficient signal output when stimulated, resulting in a low sensitivity, which severely hinders TDPF sensing applications like acoustic wave detection and deformation sensing. More sophisticated TDPF inner structures are designed to enlarge the active areas, thus improving sensing performance.

The first strategy to increase the active area is to fold the conductive polymer layer. A folded TDPF structure, using two cross-inserted “U”-shaped CPE layers to enlarge the active area that is approximately 3 times larger than that of the traditional rectangular structure, has been fabricated [73]. This folded structure could be further developed into a multi-folded structure using comb-shaped CPE layers. Another similar strategy is to adopt a spiral structure where two CPE layers and two PVDF-based mats are co-interfaced together, exhibiting an ultra-large active area [79].

## Applications

### Sensors

Piezoelectric materials convert mechanical deformations to electric signals, allowing the detection of vibrations, sound waves, forces and other mechanical displacements. Sensing is one of the major applications of TDPFs. Compared to traditional piezoelectric sensors, TDPFs are biocompatible, thin, highly flexible and wearable, which could be interfaced with tissue and organs forming innovative bioelectronics or be integrated into fabrics and textiles forming smart garments [80–82]. In this section, we will highlight the sensing function of TDPFs for intriguing applications in underwater security, audible sound detection and communication, healthcare monitoring, and space security.

#### Aquatic acoustic sensors

Aquatic acoustic sensing is acting as a key technology demanded for ocean activity monitoring, underwater constructions and ocean communications [83]. TDPFs have been exploited for underwater acoustic sensing. Compared with conventional underwater acoustic sensors based on BTO and PZT, the TDPF sensor made out of polymeric piezoelectric materials like PVDF and P(VDF-TrFE) exhibits better acoustic impedance matching with water, higher hydrostatic response, and broader bandwidth [84].

**Fig. 6:**
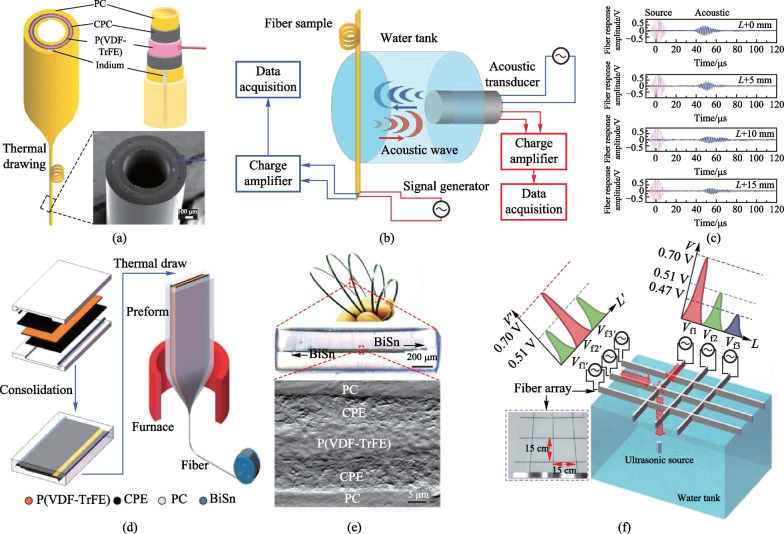
Thermally-drawn piezoelectric fibers for aquatic acoustic sensing and positioning. **a** Structure of a cylindrical TDPF for acoustic sensing. **b** Experimental set-up for acoustic characterization of piezoelectric fibers. An acoustic wave travels across a water tank from a water-immersion acoustic transducer to a fiber sample, and vice versa. **c** Temporal traces of electrically amplified acoustic signals detected by a piezoelectric fiber, shown together with the excitation signals. **d** Schematic diagram of the fabrication process of a rectangular TPDF for acoustic sensing. **e** Digital photo, optical microscope image, and the cross-section SEM image of the rectangular TDPF. **f** Schematic of an acoustic sensor network based on a 3 × 3 piezoelectric fiber array for underwater acoustic positioning. Reprinted with permission from Refs. [55, 71]

Egusa et al. first explored TDPFs’ applications in underwater acoustic sensing [55]. The fiber design adopted the traditional circular and rectangular structure. The sensing material was piezoelectric P(VDF-TrFE). CPC was used as the electrodes because it can form large areas interfacing with P(VDF-TrFE) (Fig. 6a). The TDPF fiber could efficiently sense the sound wave pulses with central frequency of 1 MHz emitted by a water immersion ultrasonic transducer (Fig. 6b, c), the response of which is shown in Fig. 6c. The measured piezoelectric response of the TDPF fiber followed the intrinsic frequency profile of the transducer, confirming the feasibility of using TDPFs as acoustic sensors.

To further extend the applicability of the TDPFs, Wang et al. developed a rectangular fiber that consisted of a rectangular P(VDF-TrFE) sheet sandwiched between two CPE conductive layers, each of which was interfaced with a BiSn electrode (Fig. 6d, e) [71]. The acoustic response of the fiber was first evaluated by measuring the output voltage when the fiber was subject to the excitement of a monochromatic ultrasound wave in a water tank. The peak-to-peak voltage *V*_app_ was detected to be larger than 100 mV, with a signal-to-noise ratio (SNR) of greater than 20 dB. The fiber could also distinguish two acoustic waves with a frequency difference down to 0.01 MHz, demonstrating an excellent detection resolution. Despite the excellent sensitivity, the single fiber could only detect the presence of the ultrasound stimulus and its amplitude because the sensing device was continuous along the entire fiber length. To achieve more complex functionality like sound localization, a 3 × 3 acoustic fiber sensor network assembled by six TDPFs was thus constructed (Fig. 6f). Measuring the output voltage of each individual fiber enabled the detection of the sound wave that was located under the fiber network. The spatial resolution of this sensor network reached up to 1 cm, suggesting great potential in underwater acoustic positioning. Thanks to its flexibility, the fiber could withstand harsh impacts and shocks in the ocean. We expect that more exquisite fiber design could enable more complex capabilities like 3D underwater positioning.

#### Audible sound detection and communication aids

**Fig. 7:**
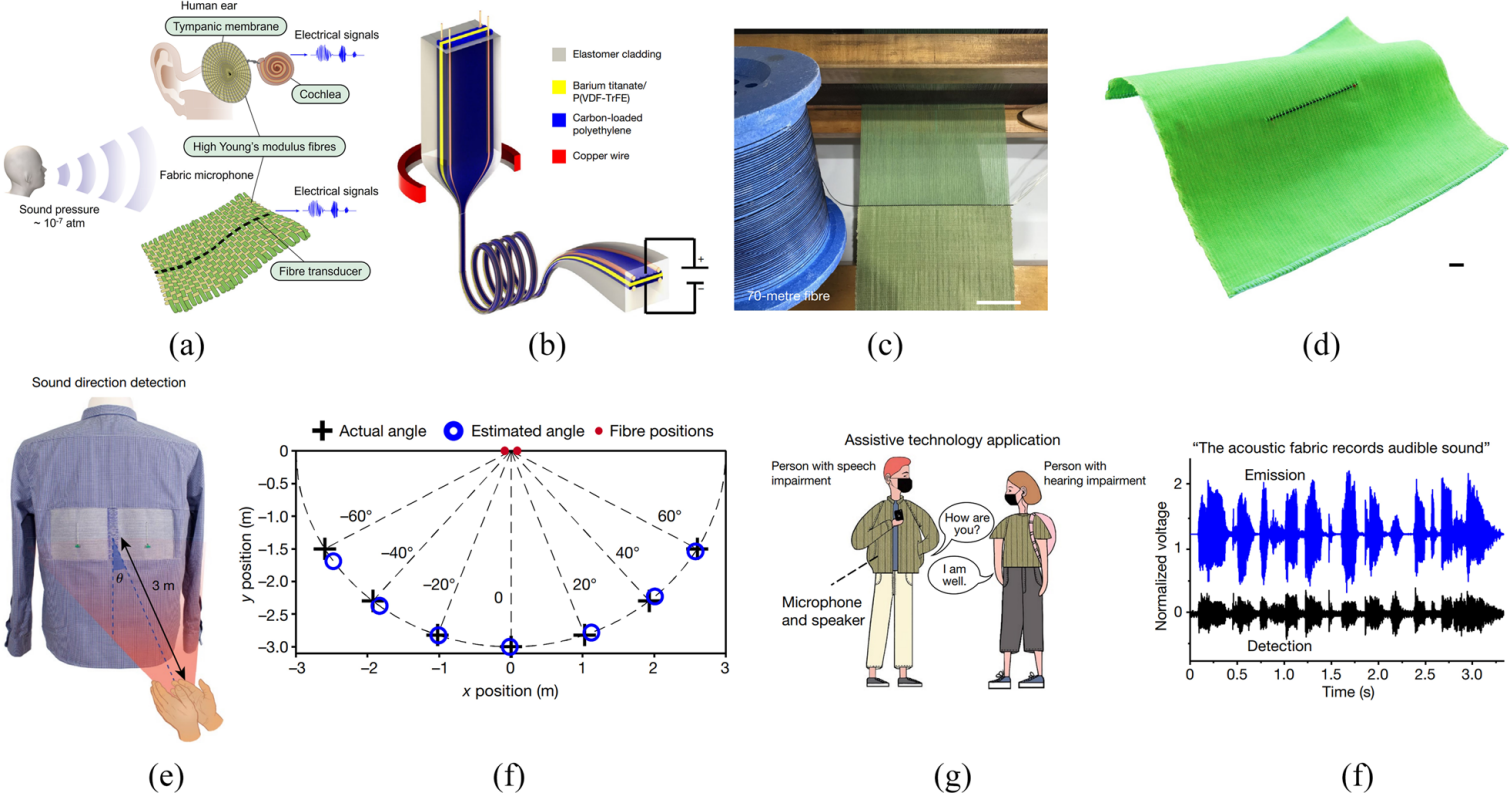
Application of TDPFs for communications. **a** Tympanic membrane structure and development of the fiber-on-membrane model. **b** Structure of a rectangular TDPF used for communication. **c** TDPFs incorporated into the fabric via weaving process. **d** Photograph of the Twaron-weft acoustic fiber containing one fiber transducer. **e** Photograph of TDPF woven acoustic fabric integrated shirts. **f** Sound direction detection performance of TDPF-based wearable electronics. **g** TDPF based acoustic fabrics could act as an assistive communication technology. **h** Demonstration of acoustic communication using two shirts. Reprinted with permission from Ref. [75]

The detection of audible sound, ubiquitous in our everyday lives, is important for speech communications, education, acoustic localization, and medical diagnosis. Although conventional TDPFs can convert mechanical vibrations to electrical signals, their poor performance in the audible frequency range precludes their use for audible sound. Recently, Yan et al. constructed an unprecedented piezoelectric fiber to address this long-lasting challenge [75, 85]. There are three unique features of the fiber. First, the piezoelectric composite is a porous ferroelectric structure that exhibits a piezoelectric coefficient *d*_31_ as high as 46 pC/N. Secondly, the fiber design is asymmetric, which enables more significant strain in the piezo material. Thirdly, the fiber cladding is soft, which concentrates the stress into the piezo domain, thus delivering a superior output voltage. Inspired by the human auditory system, the authors developed a “fabric ear” based on the piezoelectric fiber (Fig. 7a–d). The fabric could detect audible sound with performance on par with off-the-shelf rigid microphones.

This is the first time a fabric that is a sensitive sound collector and transducer has been realized, opening entirely new usage categories for fabrics. For example, a fabric with dual acoustic fibers measures the precise direction of an acoustic impulse with ~ 1° accuracy, which could be useful in assisting users to selectively hear in a noisy environment or todetermine the location of a gunshot for security applications (Fig. 7e, f). In addition, bi-directional communications are established between two fabrics working as sound emitters and sound receivers, which could facilitate efficient communication between individuals suffering from speech or hearing impairments (Fig. 7g, h). Additionally, the machine washability and mechanical deformation cyclability of this acoustic fabric are superior, as demonstrated by the stable capacitance over thousands of deformation cycles or ten machine wash cycles.

#### Healthcare sensors

**Fig. 8:**
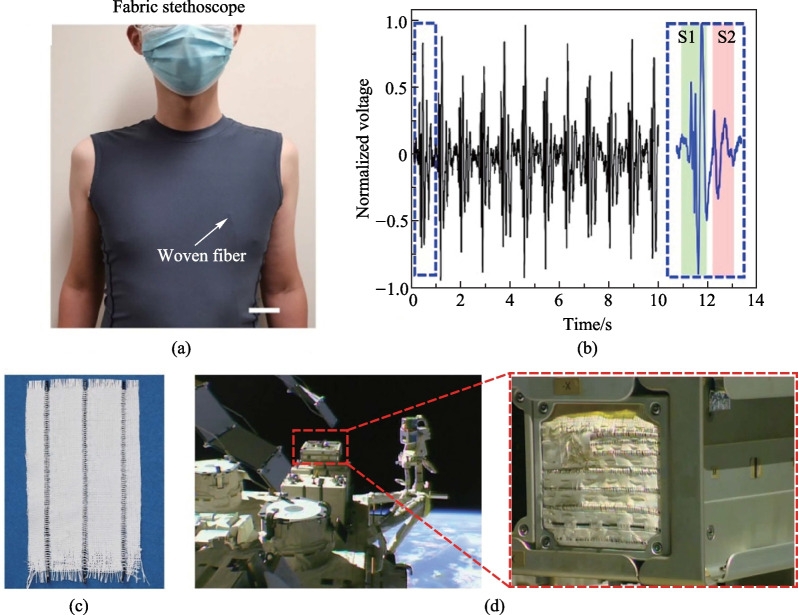
TDPF fabrics for health care and space dust sensing. **a** Photograph of the experimental scenario of the fabric stethoscope. **b** Detected cardiac signals from the fabric stethoscope. **c** A fabric containing three black TDPF sensors for dust sensing. **d** Photograph showing sensing fabrics mounted on the Exposed Experiment Handrail Attachment Mechanism (ExHAM) facility on the International Space Station (ISS). The experiment, which began in October 2020, has been studying the resiliency of different types of fabric sensors when they are exposed to the harsh environment of low Earth orbit. The amplified image shows details of the mounted fabric. Reprinted with permission from Refs. [75, 86]

Current devices and instruments for medical diagnosis are very powerful for precise measurement of physiologic signals, offering fundamental tools for modern healthcare with considerable accuracy and reliability. Such systems however are heavy and cumbersome and require professional personnel for operation and treatment, which is not suitable for continuous and long-term medical diagnosis. Wearable electronics represents an innovative technology for personalized healthcare. The “fabric ear” developed recently could act as a wearable stethoscope capable of hearing heart sound [75]. As shown in Fig. 8a and b, the shirt that integrates a piece of such fabric is interfaced with the chest and auscultates cardiac sound signals. It measured the heartbeat rate of 70 beats per min. It also detected louder sound (S1) and weaker sound (S2) with a SNR significantly surpassing the state-of-the-art thin-film acoustic devices. Imperceptibly interfaced with the human body, the acoustic fabric could enable comfortable, continuous, long-term heart and respiratory auscultation.

#### Space dust sensors

Space dust and micrometeoroids that move at a speed of up to 50 km/s, with an average speed of around 10 km/s in space, pose significant threat to astronauts. Conventional methods for the detection of space dust rely on embedding rigid and cumbersome detectors into space suits which significantly increases their weight. The acoustic fabric has been integrated into a Beth cloth, forming an unprecedented aerospace suit. It has been deployed at the International Space Station since 2020 (Fig. 8c, d). Thanks to its high sensitivity to mechanical impact, the fabric can detect the particle impingent wherever the particle strikes. The fabric maintains the same performance after staying in space for 15 months! This disruptive technology paves the way for efficient sensing of damage in space [86, 87].

### Actuators

Piezoelectric materials can also convert electrical energy to mechanical energy, acting as the key functional domain in actuators. When driven by an appropriate external electric field, TDPFs can oscillate with the same frequency as the electric field and emit modulated sound waves.

#### Underwater actuators

Underwater actuators serve as another essential component for underwater acoustic communications. For example, acoustic actuators used in underwater vehicles and scientific probes can send sensing information and research data in a wireless communication way. As opposed to planar rigid devices, TDPF actuators share the same advantageous characteristics as TDPF sensors, including high flexibility and adaptability, high sensitivity and excellent impedance matching. In Egusa’s work [55], P(VDF-TrFE) based TDPFs are also experimented with acoustic actuators to generate sound waves with central frequency of 1 MHz, a typical frequency in ultrasound imaging applications. A heterodyne optical vibrometer was used to measure the vibration of the fiber. The emitted acoustic frequency was consistent with the modulating frequency.

**Fig. 9:**
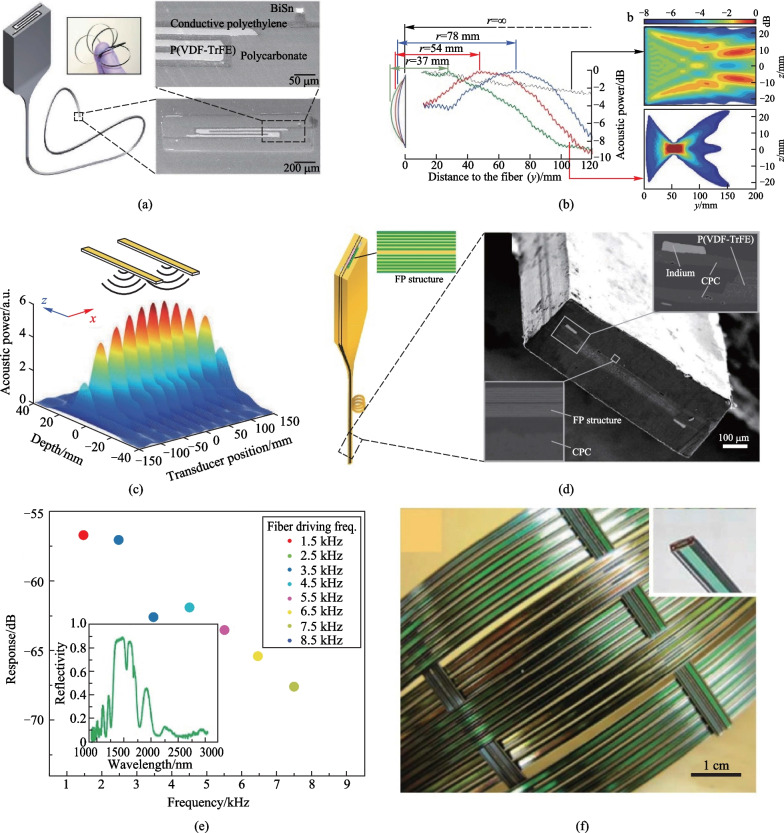
TDPFs used for actuating and modulating. **a** Structure of a rectangular TDPF for acoustic actuating and its SEM images. **b** Measured and simulated acoustic power field produced by a bent TDPF. **c** 3D representation of the acoustic pressure field of a two-fiber array emitting in phase. **d** Fabrication process and structure of an integrated piezoelectric Fabry–Pérot (FP) rectangular TDPF for optical modulation. **e** Piezoelectricity and reflection spectrum of the integrated TDPF. **f** Two-dimensional device fabric constructed by knitting the piezoelectric/Fabry–Pérot TDPF fibers as threads. Reprinted with permission from Refs. [55, 73]

In addition to single fiber actuators, acoustic fiber arrays have been designed to generate acoustic waves with complex patterns for applications including focused ultrasound surgery and ocean observation. Multiple fibers in the arrays can generate coherent acoustic waves, rendering various acoustic wave patterns. Chocat et al. fabricated TDPFs with the folded structure to increase the active area up to several-folds, which could drastically improve the energy transducing efficiency (Fig. 9a) [73]. Two-fiber and four-fiber phased arrays were assembled to generate various acoustic power patterns by altering the phase difference between the fibers, as is shown in Fig. 9c. In addition to the conventional method of beam focusing, the mechanical flexibility of TDPFs enabled a unique alternative approach. It was discovered that control over the bending radius of the fiber also permits beam focusing at a designated position (Fig. 9b). The acoustic energy could be focused on a distance roughly equal to the fiber bending radius.

#### Optical modulators

TDPFs have also been demonstrated to have the potential to modulate sophisticated optical devices. A highly integrated fiber that consisted of a fiber mirror and a piezoelectric domain was thus fabricated (Fig. 9d). The fiber mirror was essentially a Fabry–Pérot optical cavity with 90% reflectivity at 1500 nm. The piezoelectric domain was driven using a sine wave at frequencies varying from 1.5 to 8.5 kHz with an input voltage of 10 V. The vibration of the hybrid fiber was measured using heterodyne interferometry by focusing the laser beam on the Fabry–Pérot component. The side-band amplitude was measured to be distinct, demonstrating that the piezoelectric domain enhanced or dampened the optical signals [55]. The piezoelectric Fabry–Pérot fiber could be woven into fabric (Fig. 9f) for more complex functionalities and applications in optics, actuation and fashions.

### Energy harvesters

**Fig. 10:**
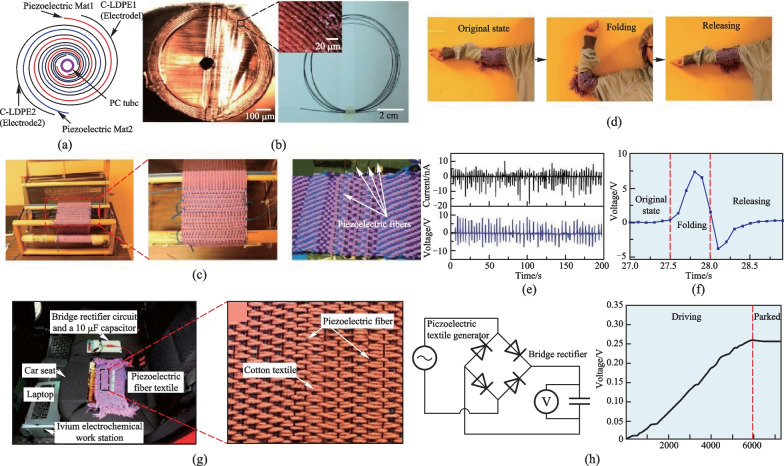
TDPF energy harvesters. **a** Structure of a spiral TDPF for energy harvesting. **b** Photo of the cross-section of the TDPF. **c** TDPFs were woven into textiles forming piezoelectric energy harvesting systems via the Dobby loom process. **d** A TDPF textile used as a prototype generator with a 90° folding–release action of the elbow. **e** Open-circuit voltages and short-circuit currents generated by the piezoelectric textile during repeated fold–release motion of the elbow. **f** Open-circuit voltage of the piezoelectric textile in a fold–release elbow action. **g** Experimental setup of an in-car test for the TDPF textile used as an automotive microgenerator pad. **h** Energy generation performance of the TDPF seat pad generator during a 6000 s driving test charging a 10 μF capacitor. Reprinted with permission from Ref. [79]

Power management of wearable electronics is crucial for their reliable operations. While traditional energy generators based on solar energy exhibit high power-supply capability, they are too heavy and cumbersome to be integrated with wearable devices. In contrast, thin and flexible TDPFs could be woven into fabrics that allow the conversion of mechanical energy originating from human motion to electrical energy, thus powering small-sized wearable electronics. Lu et al. fabricated spiral-structured TDPFs based on BTO-PVDF (20% wt BTO) and used them as primary components for small-scale prototype generators [79]. In the first generator, 4 TDPFs were woven into a textile via a classic Dobby loom process (Fig. 10a–c). The 20 cm TDPFs were connected in series to operate as a prototype generator, and the textile was wrapped around the human elbow. The 90° bend and release of the elbow generated an open-circuit voltage of up to 10 V and short-circuit current of 5 − 15 nA (Fig. 10d–f). The high voltage output of this TDPF generator was attributed to the large active area of the spiral structure. A second prototype TDPF generator was prepared by weaving 15 parallel connected TDPFs into a seat pad of vehicles. When the passenger sat on the seat pad, the vibrations were directly converted into electrical signals. During a 6000 s driving experiment with a 22 kg sandbag placed on the pad, the generated electrical energy charged a 10 μF capacitor from 0 to 0.3 V (Fig. 10g–i), illustrating the possibility of adopting TDPF textiles for small-scale convenient energy generation.

## Conclusion and outlook

Thermally-drawn piezoelectric fibers are emerging as an innovative class of flexible and wearable technology with potentials in marine and aerospace security, personalized and precision healthcare, speech sensing and communication, and energy harvesting. In this review, we highlight recent advances of thermally-drawn piezoelectric fibers, including control over material microstructure, architecture design, device fabrication and applications. Driven by recent scientific and technological breakthroughs in materials and fabrication, thermally-drawn fibers have grown more sophisticated. Nevertheless, some challenges relevant to fundamental and applied science remain.

Piezoelectric materials: Compared to typical ceramic piezoelectric materials, the polymeric piezoelectric materials exploited in the thermal-drawing platform possess limited piezoelectric properties and performance, which restricts the real-world application of the fiber. A recent innovation in control over the material microstructure at the nanoscale level has demonstrated a unique and powerful methodology for the fabrication of unprecedented fiber devices [75]. In addition, newly-developed high-performance polymeric piezoelectric materials can be harnessed to develop innovative fiber devices [88].

Fiber architecture: Thermally-drawn fibers are transitionally symmetric, and the in-fiber structure is continuous along the entire fiber length in general, which limits their use for many applications. Breaking the translational symmetry of the current piezoelectric fibers will enable unparalleled applicability and opportunities. More fiber processing methods should be combined with the thermal-drawing technique to break the roadblock.

Real-world application: Material cost, fabrication simplicity, device reproducibility and manufacturing scalability are the keys to the commercialization of thermally-drawn fibers. Even though thermal drawing offers unparalleled scalability for large-scale fiber manufacturing, the current piezoelectric materials are relatively costly, and the preform fabrication approaches involve time-consuming hand operation. More innovations in raw materials and fabrication methodologies are required to improve the potential for commercialization.

Thermally-drawn piezoelectric fibers are rapidly evolving as a multidisciplinary material. With continued and concerted efforts in materials and fabrication, control over microstructure and architecture, and device integration, more advanced fiber-shaped flexible and wearable piezoelectric electronics with more sophisticated functionalities and practical applications will emerge to deliver more value to society. Watch out for the technology!
